# Use of #SaludTues Tweetchats for the Dissemination of Culturally Relevant Information on Latino Health Equity: Exploratory Case Study

**DOI:** 10.2196/21266

**Published:** 2021-03-01

**Authors:** Amelie G Ramirez, Rosalie P Aguilar, Amanda Merck, Cliff Despres, Pramod Sukumaran, Stacy Cantu-Pawlik, Patricia Chalela

**Affiliations:** 1 Department of Population Health Sciences Institute for Health Promotion Research UT Health San Antonio San Antonio, TX United States

**Keywords:** Latino, social media, social cognitive theory, health communication, health equity, policy, community health, mobile phone

## Abstract

**Background:**

Latinx people comprise 18% of the US adult population and a large share of youth and continue to experience inequities that perpetuate health disparities. To engage Latinx people in advocacy for health equity based on this population’s heavy share of smartphone, social media, and Twitter users, *Salud America!* launched the #SaludTues Tweetchat series. In this paper, we explore the use of #SaludTues to promote advocacy for Latinx health equity.

**Objective:**

This study aims to understand how #SaludTues Tweetchats are used to promote dissemination of culturally relevant information on social determinants of health, to determine whether tweetchats serve to drive web traffic to the *Salud America!* website, and to understand who participates in #SaludTues Tweetchats and what we can learn about the participants. We also aim to share our own experiences and present a step-by-step guide of how tweetchats are planned, developed, promoted, and executed.

**Methods:**

We explored tweetchat data collected between 2014 and 2018 using Symplur and Google Analytics to identify groups of stakeholders and web traffic. Network analysis and mapping tools were also used to derive insights from this series of chats.

**Results:**

We conducted 187 chats with 24,609 reported users, 177,466 tweets, and more than 1.87 billion impressions using the hashtag #SaludTues during this span, demonstrating effective dissemination of and exposure to culturally relevant information. Traffic to the *Salud America!* website was higher on Tuesdays than any other day of the week, suggesting that #SaludTues Tweetchats acted effectively as a website traffic–driving tool. Most participants came from advocacy organizations (165/1000, 16.5%) and other health care–related organizations (162/1000, 16.2%), whereas others were unknown users (147/1000, 14.7%) and individual users outside of the health care sector (117/1000, 11.7%). The majority of participants were located in Texas, California, New York, and Florida, all states with high Latinx populations.

**Conclusions:**

Carefully planned, culturally relevant tweetchats such as #SaludTues can be a powerful tool for public health practitioners and advocates to engage audiences on Twitter around health issues, advocacy, and policy solutions for Latino health equity. Further information is needed to determine the effect that #SaludTues Tweetchats have on self- and collective efficacy for advocacy in the area of Latino health equity.

## Introduction

### Inequities in Latinx Health

In the United States, Latinx people comprise over 18% of the adult population and over a quarter of youth, and they continue to face vast health disparities in obesity, diabetes, heart disease, and certain cancers [1-5]. These disparities are largely the result of systemic inequities in housing, education, employment, health care, and transportation, which disproportionately burden communities of color with unstable housing, poor quality schools, unfair wages, unsafe streets, unhealthy food, numerous other barriers to opportunity, and poorer health outcomes [6,7]. Reducing disparities in health requires advocacy and policy solutions to improve social and environmental factors and create more equitable living conditions [8].

The internet is an important source for distributing information to promote advocacy and policy solutions. More specifically, digital content curation, a systematic, refined process to create tailored online and social health information for an audience, provides an opportunity to empower Latinx social media users to advocate for policy changes [9]. Trends show that 90% of Latinx people aged younger than 50 years are connected online, including high rates of smartphone ownership and engagement with social media [10-13].

Approximately 80% of Latinx people own a smartphone and use it to access the internet [11,14,15]. Latinx people also lead in the use of Twitter (25% vs 24% of non-Latinx Black people and 21% of White people), spending approximately 6 hours a day on social media, which enables social interaction, self-expression, and consumption of news and information [16]. We aimed to reach Latinx social media users as well as health advocates by developing a continuous stream of evidence-based stories, research, resources, and policy change solutions to promote Latinx health equity.

### Tweetchats for Health Advocacy and Health Promotion

Tweetchats are social media events that can be used to engage communities and partners in sharing health information [17-20]. Many public and private entities have used Twitter hashtags and tweetchats to promote awareness of a topic, elevate campaigns, share news and updates, drive political participation, and promote conference engagement [17,18,21-29]. For example, a number of local public health departments use Twitter to communicate public health messages as part of specific public health campaigns [17,30]. However, little is known about the effectiveness of using tweetchats to engage audiences, particularly around Latinx health equity and advocacy topics. With this in mind, *Salud America!* launched the #SaludTues Tweetchat series to complement ongoing digital content curation and social media tactics to raise awareness about Latinx health equity issues and solutions.

*Salud America!* (based at UT Health San Antonio) is a national Latinx-focused organization with an online network of over 400,000 community leaders and advocates, parents, school personnel, health care workers, and researchers with an interest in Latinx health [31]. The team behind *Salud America!* uses digitally curated, culturally relevant, and research-based stories, videos, tools, and events such as tweetchats to inspire and support healthy changes to policies, systems, and environments [9].

The team’s digital content curation is influenced by the Social Cognitive Theory (SCT) by Bandura [32,33]. SCT guides the creation of peer-modeled blog posts and social media messages that inspire community action to start or support social change. At the population level, *Salud America!*’s dissemination approach is largely guided by a combination of SCT and Diffusion of Innovations Theory [32,34]. According to Bandura, adoption of social innovations, such as policy solutions to improve health equity, can be achieved through mass communications coupled with symbolic modeling [32]. This produces a dual path of communications in which both direct exposure to digital content and mediated connections to individuals through social media may influence personal and social change and prompt advocacy [32]. For example, within the context of a Tweetchat, participants may advocate for healthier environments by sharing examples of policy solutions and encouraging others to take local action. Initially, some social media users may be slow to advocate for policy solutions, but as time progresses and more individuals model positive change by sharing policy solutions on social media, the rate of acceptance accelerates [34,35].

In fall 2014, *Salud America!* established a weekly #SaludTues Tweetchat series using its Twitter handle (@SaludAmerica) [9,32,33]. The goals of these chats are to raise awareness about the social and environmental inequities that contribute to disparities in Latinx health and inspire policy solutions and advocacy actions by posing questions and providing positive verbal persuasion, stories, and resources to build self- and collective efficacy. To accomplish this, *Salud America!* disseminates digitally curated content on Latinx health equity from its website [36]. In this case study, we examine data from a series of 2014 to 2018 #SaludTues Tweetchats collected via Symplur, a social media analytics platform for health care [37]. We aim to provide a step-by-step guide on how to organize a successful tweetchat and also describe lessons learned by outlining the process that *Salud America!* uses for organizing its weekly Latino health advocacy–oriented tweetchats. The primary objectives of this paper are as follows. Specifically, we aim to explore the following:

- How are #SaludTues Tweetchats used to promote the dissemination of culturally relevant information on social determinants of health?
- Do tweetchats serve to drive web traffic to the *Salud America!* website?
- Who participates in #SaludTues Tweetchats and what can we learn about the participants?

## Methods

### The Salud America! Approach to Hosting Tweetchats

The following analysis does not involve human research and was therefore exempt from an institutional review board approval. We begin with a description of our approach to conducting tweetchats (objective 1). *Salud America!* hosts a 60-min #SaludTues Tweetchat on Twitter from 1 to 2 PM EST every Tuesday. This date and time were selected based on Twitter engagement metrics at the time and to avoid competing with other popular tweetchats such as #FoodFri, a popular tweetchat that takes place between 1 and 2 PM EST on Fridays [36]. Although not everyone may be available to participate during this time, we found that midday chats work well for engaging users and cohosts across different US time zones. Tweetchats are accessible to anyone on Twitter by following the #SaludTues hashtag.

Three constructs derived from SCT—the theory behind our digital content curation—are relevant to #SaludTues: (1) peer role modeling, (2) positive verbal persuasion, and (3) perceived self-efficacy [33]. The first two of these constructs were employed to prepare each week’s tweetchat materials, with the goal of increasing a person’s self-efficacy to engage in message sharing and promoting advocacy for Latinx health equity [9]. For instance, we might prepare an answer tweet with a message about how one of our Salud Heroes (role models) helped promote equity in their community, in response to a question that asks, *how can we advocate for change?* The tweet would include a positive message about the hero, a link to the full-length story, an image of the hero, and perhaps an emoji such as a flexed arm to signal strength and confidence. If the role model is relatable and the message is motivational, the person may experience an increase in self-efficacy for engaging in advocacy actions both online and potentially offline. The Social Ecological Model was also applied to craft messages that help participants understand how multiple dimensions of social and environmental factors influence health and encourage participants to promote social change [38,39]. In addition, the Transtheoretical Model of Behavior Change, also known as the *Stages of Change* model, was used during chats to identify tweetchat participants’ levels of readiness for action and to craft messages in response to this.

#### Planning and Scripting Questions

The *Salud America!* communications team selects weekly tweetchat topics that align with Latinx health equity issues and campaigns. Some topics are specific to *Salud America!*’s content curation scope, which currently focuses on healthy families and schools, healthy neighborhoods and communities, and healthy and cohesive cultures, whereas other topics are based on weekly and monthly observances and partner requests. For example, in February 2017, the tweetchat on *Our Heart Loves Physical Activity* observed National Heart Month at the request of the US Department of Health and Human Services’ Office of Minority Health. A #SaludTues Tweetchat in October 2017 honored National Hispanic Heritage Month and a tweetchat in November 2017 promoted the release of *Salud America!’s Early Childhood Development and Latinx Kids Research Review*. Another tweetchat hosted in October 2018 explored issues and solutions to the lack of Latinx people in clinical trials.

*Salud America!* serves as a *host* for each #SaludTues chat [40] and invites organizations and individuals to serve as *cohosts*. The criteria for selecting cohosts vary but can usually be categorized into one of the following: (1) a subject-matter expert or group, (2) an active advocate or advocacy group, and (3) users that can offer a unique perspective. It is also helpful to find out whether a cohost retweets regularly, if their tweets are culturally sensitive, and whether they typically share engaging tweets that contain images, URL links, and useful media, as these are more likely to be retweeted. Although *Salud America!* seeks to share content from their websites, cohosts are not expected to have a website. Cohosts are asked to: (1) review tweetchat questions and prepare 1-3 responses in advance; (2) promote the event during the week before the Tweetchat; and (3) actively engage during the 60-min Tweetchat by tweeting, retweeting, liking, and replying to other contributors in real time.

As the host, *Salud America!* drafts 6-8 questions and 12-24 responses each week. The first few questions ask participants to consider, define, or share personal experiences with the topic to build relevance and salience. Then, questions implore exploration of evidence, scientific research, and anecdotes of how the topic relates to Latinx health through a social and environmental lens rather than just an individual lens. Finally, questions focus on policy solutions or advocacy actions to address or improve social and environmental factors.

**Figure 1:**
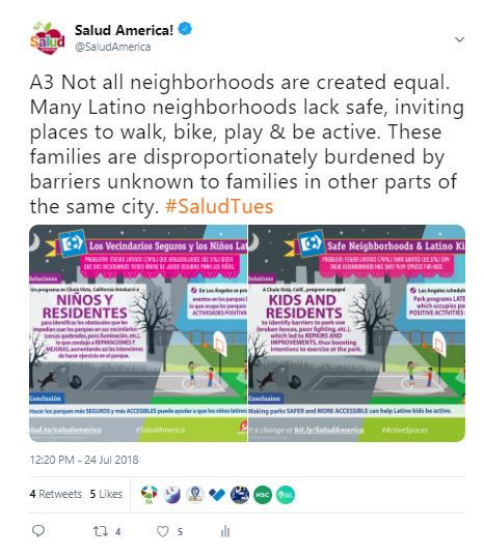
Example of a tweet during #SaludTues Tweetchat.

*Salud America!* prepares responses to the questions with links to digitally curated content from the *Salud America!* website, such as educational resources, stories of peer models, and specific opportunities to take action as well as culturally appropriate photos, graphics, and infographics, often derived from our research reviews. For example, Figure 1 shows a tweet response with an English and Spanish infographic from our research review on safe neighborhoods. *Salud America!* also prepares template retweets and replies to quickly engage with cohosts and participants. 

Similar to a real-life discussion, *Salud America!* works to share, echo, and expand on evidence, redirect focus away from individual behavior change to systemic change, and encourage participants to seize opportunities for advocacy. We offer some examples in the following section.

#### Promotion and Real-Time Participation

As a host, *Salud America!* drafts promotional materials each week, including a blog post, sample tweets, and graphics. The week before each Tweetchat, *Salud America!* promotes the chat on Twitter at least once a day, escalating to several times a day, the day before, and the day of the chat. *Salud America!* tags 10 individuals and organizations in each promotional graphic to encourage wider sharing and participation. To identify to-be-tagged individuals and organizations, *Salud America!* searches tweets and profile bios for various keywords relevant to the specific topic. *Salud America!* scrolls through their feed for diverse perspectives on health disparities, social inequities, and policy solutions. For example, a tweet promoting a tweetchat on how to systemically promote the health benefits of swimming tagged 2 individual swim safety advocates, 2 swim safety foundations, 1 drowning prevention coalition, 1 physical activity coalition, and others. As policy solutions do not occur in a vacuum, it is important to expand promotional efforts beyond Latinx people to include various policy and health equity stakeholders.

Although following the *latest* stream of #SaludTues content on Twitter, the host and cohosts read each tweet and respond accordingly while also tweeting out prescripted responses. On the basis of participants’ tweets, the team gauges the level of awareness and readiness for action similar to a real conversation. For example, if a participant appears surprised by the data or unsure about the connection between health disparities and inequities in social and environmental factors, *Salud America!* crafts a response to raise awareness. Similarly, if a participant appears informed and passionate, *Salud America!* crafts a response to build self- and collective efficacy and to inspire action to address social inequities. When crafting replies, *Salud America!* quickly decides whether to encourage more learning by sharing links to research reviews and other informational content on the *Salud America!* website; to inspire action and include links to Salud Hero stories and other examples of policy change on the *Salud America!* website; or to provide positive reinforcement for dissatisfaction with and commitment to addressing social and environmental inequities. The Tweetchat continues for an hour and ends with sending thank you tweets and information on how to engage further with the *Salud America!* network.

#### Collecting Tweetchat Metrics

All Tweetchats are archived via online software (Wakelet) [41]. These archives capture tweets with the hashtag #SaludTues and are sharable *recaps* that can be accessed by those who miss the chat. For capturing metrics and conducting routine data analysis, the *Salud America!* communication team used Symplur Signals (Symplur LLC) online software to capture metrics for each chat [37]. Symplur Signals is a paid subscription analytics platform that links directly to the Twitter application program interface [42]. Only tweets with the #SaludTues hashtag are included in the metrics. Metric data includes the number of participants, tweets, and impressions (tweets per participant with hashtag multiplied by a user’s number of followers at that time=impressions). After each chat, *Salud America!* compiles Symplur data (total tweets, impressions, users, word bubbles, top tweeters, most retweeted, most mentioned, etc) into a PowerPoint report to share with cohosts.

### Data Analysis for 2014-2018 #SaludTues Chats

Descriptive statistics for tweetchats, such as means and totals per month and year, were tracked on a spreadsheet. To determine whether #SaludTues Tweetchats serve as a funnel from Twitter to the *Salud America!* website (objective 2), we used Google Analytics (GA) data to gauge website usage on different days of the week during the first year of our comprehensive website launch (September 1, 2017, to August 31, 2018) [43]. The bulk of the remaining analysis is focused on understanding who participates in #SaluldTues Tweetchats as well as where they are tweeting from (objective 3). We also aimed to learn about the terms that were used most during chats.

**Figure 2:**
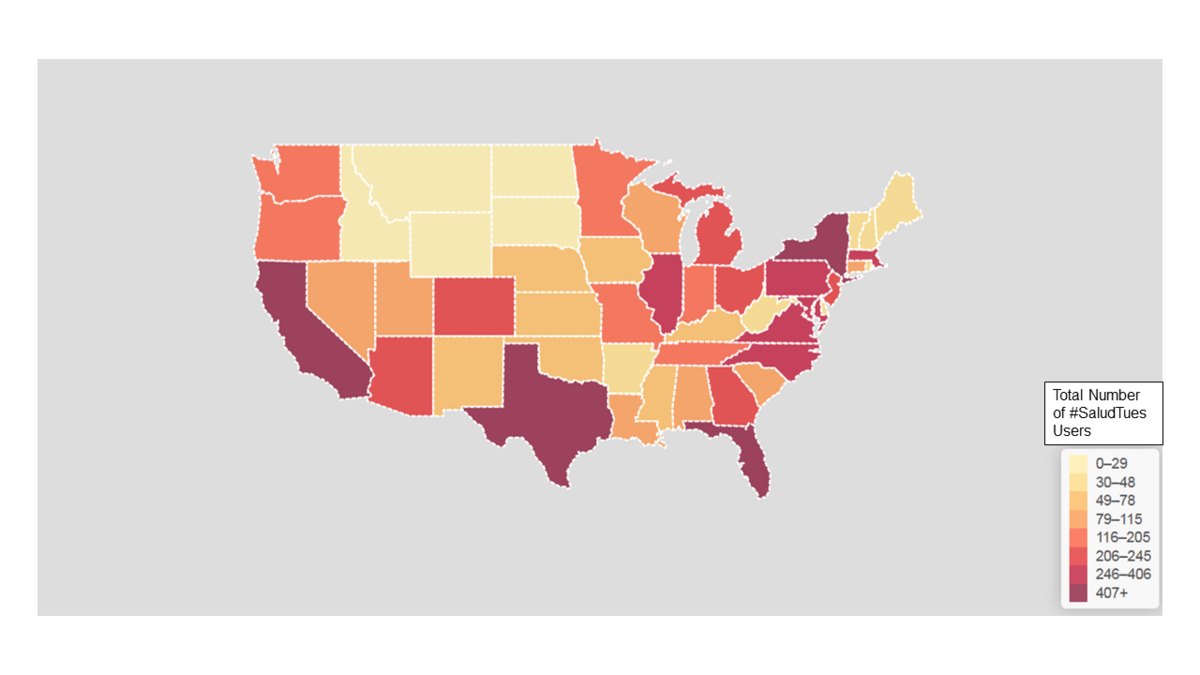
Choropleth map of the United States showing where Tweetchat users are tweeting from, between November 1, 2014, and August 31, 2018.

To understand the geographic exposure of tweetchats, a choropleth map of the United States was accessed through Symplur to determine where #SaludTues users tweet from (Figure 2). This map comprises data collected from users’ manually entered locations from their Twitter profiles (disclosing location is voluntary on Twitter).

To illustrate the variety of #SaludTues trending terms, *Salud America!* used Symplur to create 2 bubble chart visualizations of 4 tweetchats occurring in April 2018 and all tweetchats occurring over a 4-year period. Symplur Signals contains an algorithm that identifies the 500 most significant and trending terms in any health care conversation and produces a series of bubbles [44]. The size of bubbles is proportional to the frequency of words appearing in tweetchats (color only aids in differentiation) [44]. As #SaludTues Tweetchats cover a wide variety of topics on Latinx health equity, we chose to examine 1 month of data to gain insight into how topics differ between the two periods.

To understand what type of health care stakeholders participate in tweetchats, *Salud America!* relied on Symplur’s health care categories. Symplur software—using publicly available data (Twitter bios and self-identification) and proprietary algorithms combined with human evaluation and quality control—categorizes each Twitter user by their specific role in health care. Users that Symplur cannot identify are classified as *unknown*.

Taking a more traditional look at network analysis, *Salud America!* used Symplur’s visualization function to examine 1 month of #SaludTues Tweetchats (from April 2018). Symplur enables the creation of an interactive network analysis of Twitter users most central to conversations by hashtags, the size of their nodes, and how the conversation flows between them and others [45]. Nodes represent Twitter users in a community, and edges indicate direct communications between users. The larger the node, the more frequently mentioned the user [45].

## Results

### Findings From #SaludTues Tweetchats 2014-2018 and Website Traffic

The results showed that of the 187 chats between 2014 and 2018, the #SaludTues hashtag was used by 24,609 users in 177,466 tweets, garnering more than 1.87 billion impressions (Table 1). Approximately one-third of tweets collected over the 4-year period were promotional tweets leading up to the chat or occurring after the live event.

**Table 1:** Tweetchat metrics on tweets, users, and impressions collected via Symplur 2014-2018.

Category	Continuous data collected for #SaludTues	Cumulative data from weekly chats for #SaludTues	Promotional tweets for #SaludTues
Total tweets	177,466	125,541	51,925
Retweets	111,491	76,071	35,420
Tweets with links	60,515	39,962	20,553
Tweets with media	48,746	26,779	21,967
Tweets with mentions	141,051	94,902	46,149
Tweets with replies	11,310	10,450	860
Users	24,609	16,952	7657
Impressions	1,870,000,000	1,491,416,579	378,583,421

The table compares 2 methods of collecting data via Symplur. The first column represents continuous data collected from the hashtag between November 1, 2014 and August 31, 2018. The second column represents the sum of data solely for Tuesday chats during the same dates (90-min time frame [11:45 AM-1:15 PM CST]). The difference (column 3) primarily comprises promotional tweets sent before or after tweetchats. During 2017-2018, following the relaunch of *Salud America!*’s website, 24.28% (2020/8318) of *Salud America!* website users visited on Tuesday (the day of #SaludTues chats), which is the highest day of web traffic, any day of the week (Table 2), according to GA. Multimedia Appendix 1 shows a randomly chosen month of 4 #SaludTues Tweetchats in April 2018. During these chats, 292 users participated, generating 2,216 tweets and 26.7 million impressions.

**Table 2:** Daily referral traffic (Salud America! website users) from Twitter (2017-2018); N=8318.

Day of the week^a^	Count, n (%)
Sunday	463 (5.57)
Monday	1293 (15.54)
Tuesday	2020 (24.28)
Wednesday	1458 (17.53)
Thursday	1431 (17.20)
Friday	1166 (14.02)
Saturday	487 (5.86)

^a^Data collected from Google Analytics from September 4, 2017, to August 31, 2018.

These chats demonstrate a snapshot of the variety of #SaludTues metrics, the breadth of topics and cohosts, and the use of Twitter as a funnel to *Salud America!*’s website and theory-based health equity content. For the same month, the GA results showed that 33% of the traffic acquired from social media channels came directly from Twitter. Exposure to #SaludTues was global but highest in the United States. The map seen earlier (Figure 2) shows that participation was greatest in US regions with higher Latinx populations, including California (15,574,882/39,512,223, 39.42%), Texas (11,524,842/28,995,881, 39.75%), New York (3,749,257/19,453,561, 19.27%), and Florida (5,663,629/21,477,737, 26.37%).

### #SaludTues: Trending Terms

**Figure 3:**
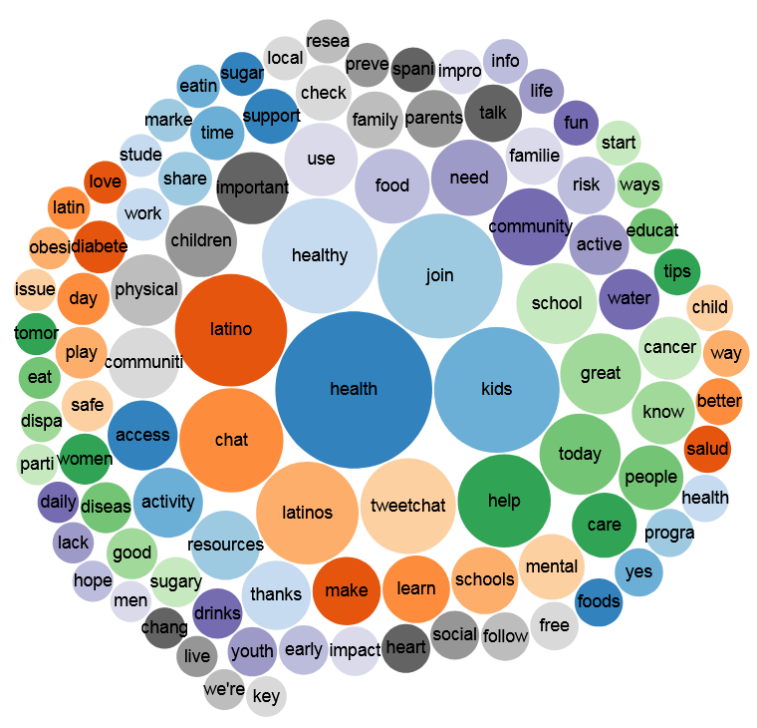
A bubble chart visualizing the 100 most frequently used words in tweets with the hashtag #SaludTues between November 1, 2014 and August 31, 2018.

Some of the most used key terms found in tweetchats were “Latino,” “Latinos,” “health,” “healthy,” and “tweetchat” (Figure 3). Additional key terms individually or in combination offer a glimpse into past and current *Salud America!* topic areas (social support, community health, mental health, safe places to play, access to care, etc) and other points of emphasis for Latinx health (cancer, diabetes, obesity, etc).

**Figure 4:**
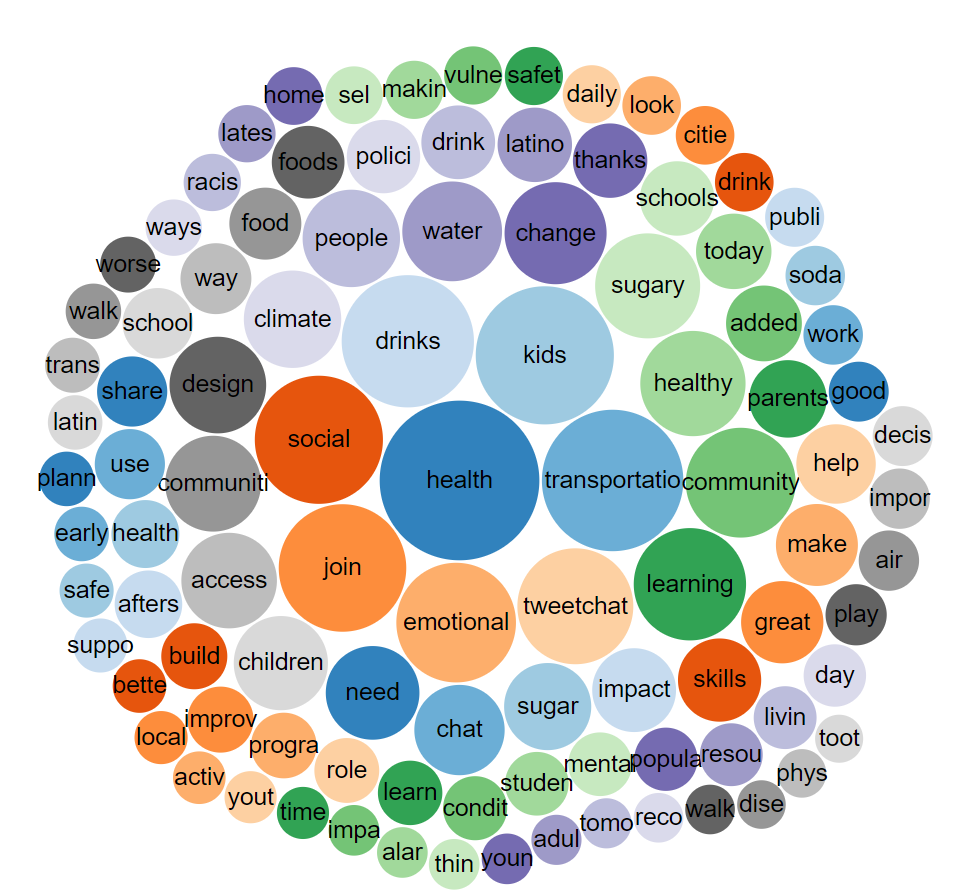
A bubble chart visualizing the 100 most frequently used words in tweets with the hashtag #SaludTues between April 1 and 30, 2018.

Figure 4 shows words specific to 1 month of #SaludTues hosted on the following topics: access to healthy foods and drinks, health and safety at home, climate and transportation, and social and emotional learning.

The majority of #SaludTues stakeholders were found to be advocacy organizations (16.5%) and other health care organizations (groups fulfilling roles in health care but not providing direct clinical care). Approximately 15% of users were classified as unknown by Symplur’s segmentation tool (Table 3). Upon closer examination of these unknown users, it appears that many are individual health advocates working outside of the health care industry in public health, whereas other accounts appear to belong to specific health campaigns (ie, Shape Up San Francisco), policy and advocacy organizations (ie, Prevent Child Abuse America), coalitions (ie, the Partnership for Active Transportation), nonprofits (ie, The Texas Hunger Initiative, Forward Promise), and some small business owners (personal trainers, bloggers, counselors, and dietitians). A few of the accounts marked as unknown did not contain a profile description. The rest of the participants were classified as individual nonhealth-related users (in nonclinical roles), nonhealth organizations, individuals from fields outside of health, government organizations, health care professionals, research and academic institutions, doctors (MDs, DOs, and PhDs who bill directly for services), provider organizations, patient advocates, caregivers, PhD researchers and academics, journalist groups, media organizations, and spam. Multimedia Appendix 2 shows a list of definitions for each segmentation category used by Symplur and seen in Table 3 [46].

**Table 3:** Top health care stakeholders by tweets for 1000 #SaludTues participants between November 1, 2014, and August 31, 2018 (N=1000).

Health care stakeholder	Count, n (%)
Doctor	33 (3.3)
Health care provider	37 (3.7)
Patient advocate	17 (1.7)
Caregiver	16 (1.6)
Researcher/academic	11 (1.1)
Journalist/media	7 (0.7)
Individual other health	80 (8)
Individual nonhealth	117 (11.7)
Organizational provider	24 (2.4)
Organizational research/academic	34 (3.4)
Organizational government	60 (6)
Organizational advocacy	165 (16.5)
Organizational media	1 (0.1)
Organizational other health care	162 (16.2)
Organizational nonhealth	89 (8.9)
Spam	1 (0.1)
Unknown	147 (14.7)

### #SaludTues: Network Analysis

**Figure 5:**
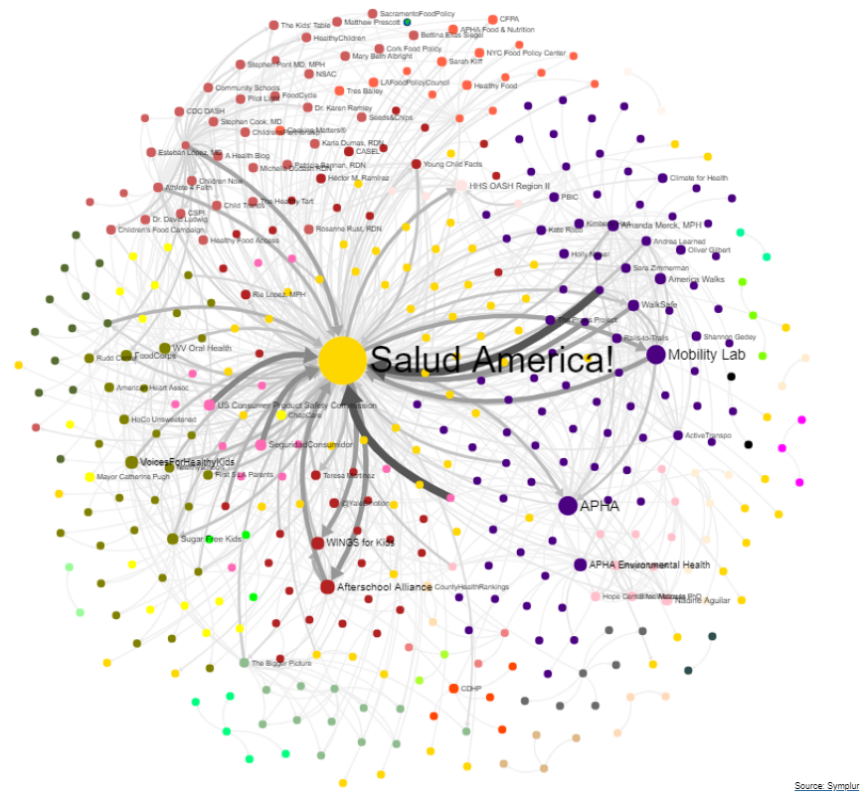
A schematic representation of the network analysis map showing 1000 participants in 4 #SaludTues Tweetchats in April 2018.

Results of the network analysis revealed that chat host @SaludAmerica is represented as the largest node (circle in the graph), meaning it is one of the most influential profiles within this network (Figure 5). Other nodes are influential health advocates and groups who have cohosted or participated in one or more tweetchats, such as governmental health agencies, health advocacy groups, physician advocates, and Latinx-focused organizations.

Symplur’s network analysis, which uses the Louvain algorithm to detect communities, identifies groups by node color [45,47]. These communities parallel the 4 distinct tweetchats, with cohosts as key authorities. From left to right, the olive-green community parallels cohosts and users from #SaludTues on April 3, 2018 (How to Improve Access to Healthy Foods/Drinks); dark pink on April 10 (Health and Safety at Home for Latinx Kids); red on April 24 (Our Kids & the Need for Social and Emotional Learning); and indigo on April 17 (Climate Changes Health: Transportation & Community Design). The light pink community in the lower right parallels cohosts and users promoting the hashtag on May 1 (Racism and its Alarming Impact on Mental Health). According to Symplur’s user guide, edges (arrows) in the network analysis represent either a reply, mention, retweet, or a quote tweet [48]. The user with the highest amount of in-degree communication, denoted by edges pointing toward a particular user, was @SaludAmerica. However, @SaludAmerica also participated in a great deal of out-degree communication, as represented by arrows pointing away from @SaludAmerica. The thickness of lines in this graph represents a higher degree of communication between the 2 groups. The West Virginia Oral Health Coalition, WalkSafe, Mobility Lab, Health and Human Services Office of the Assistant Secretary for Health Region II, The Praxis Project, The Afterschool Alliance, US Consumer Product Safety Commission, Seguridad del Consumidor, and ChapCare appeared to have had some of the highest degrees of communication with @SaludAmerica during April 2018.

## Discussion

### Principal Findings

For nearly 4 years, *Salud America!* successfully built an online platform to engage partners and audiences in tweetchats to raise exposure to Latinx health equity issues, awareness, and action opportunities. These weekly social media events complemented *Salud America!*’s existing digital content curation and provided an additional opportunity to disseminate content to enhance self-efficacy and inspire health advocacy. During tweetchats, we engaged people with specific research, resources, and stories of health and social issues, relevant to Latinx people interested in or new to health promotion and community action. We also tweeted peer-modeled case studies from across the country, demonstrating the step-by-step process to achieve a specific policy or system change. Throughout these chats, messages were amplified through frequent retweets and the live conversation that took place.

Our analysis also shows that we were successful in engaging a diverse pool of stakeholders from various backgrounds and professions in tweetchats related to Latinx health equity. Engaging diverse users in conversations related to Latinx health equity may be especially important for promoting grassroots advocacy and ultimately the development of public policy aimed at promoting Latinx equity [8,49,50]. Therefore, tweetchats may be of interest in prompting a greater level of grassroots advocacy among Latinx communities; however, further research in this area is needed.

Although the data presented here are specific to #SaludTues Tweetchats, others seeking to establish a strong base of health advocates on Twitter may benefit from adopting similar techniques to those used by our health communications team. To track progress over time and gain additional insights into one’s primary audience, it is important to regularly collect metrics on tweetchats. Organizers should start by collecting baseline website metrics before launching a regularly occurring tweetchat. It is also helpful to compare tweetchat metrics from multiple chats over a given period, as described in this paper.

### How Are #SaludTues Tweetchats Used for Promoting Dissemination of Culturally Relevant Information on Social Determinants of Health?

Tweetchat questions engaged cohosts and contributors to think critically about health and social issues by using research and examples applicable to contributors’ own experiences, such as the aforementioned Salud Hero peer model stories and videos. Tweetchat hosts and cohosts addressed the latest findings and best practices regarding the social determinants of health while encouraging contributors to share their experiences, successes, and actions. We believe our #SaludTues Tweetchats led to a positive exchange of information among chat contributors, showcasing how to more effectively craft health messages and communicate scientific, technical, or social content in concise statements while also serving as reinforcement of positive verbal persuasion to raise awareness and take action beyond the individual level. In addition, the choropleth map shows close alignment with our target audience, the Latinx population, and the areas where most Latinx people in the United States live [51]. The network analysis visualization provides interesting findings that indicate effective broadcasting and exposure of content and messages to multiple partners.

### Do #SaludTues Tweetchats Drive Traffic to the Salud America! Webpage?

Twitter is one of the highest drivers of social media traffic to the *Salud America!* website. Findings from our exploration of tweetchat data and webpage visits over 1 year showed that website traffic from Twitter was at least 7% higher on Tuesdays than on any other day of the week. This suggests that in addition to exposing thousands of Twitter users to *Salud America!* content, #SaludTues Tweetchats also helped funnel more people to our website. As digital content curation is one of *Salud America!*’s essential programmatic features, website traffic is an important metric.

### Who Participates in #SaludTues Tweetchats and What Can We Learn About Participants?

Through our exploration of tweetchat data, we found that we are reaching users from our target audience of Latinx social media users and health advocates. Over 33% of the top 1000 participants were advocacy organizations or other health care organizations. However, approximately 15% of participants were classified as *unknown*. Upon further exploration, we learned that most users classified as *unknown* appeared to be individual or community health advocates (ie, parents, teachers, employers, etc), nonprofits, campaigns, coalitions, and small businesses, all of whom remain very relevant to our work and also play an important role in inspiring policy and system change for health equity. Although we aim to reach as many Latino social media users as possible, our main goal with #SaludTues chats is to amplify information about Latino health equity via Twitter and to offer a Latino health perspective, where there might not otherwise be one.

The #SaludTues network analysis found that *Salud America!* is the central node in the figure, which is expected because the host *Salud America!* tweets out all questions and makes a concerted effort to engage with other participants during the chat. In addition, participants often engage in replying to and retweeting #SaludTues questions, which adds to higher in-degree communication occurring toward @SaludAmerica and an increased degree of centrality of the @SaludAmerica account within the #SaludTues network analysis. Similar results were seen in a previous network analysis conducted by Gomez-Vasquez et al [52], which found that *Salud America!* participates in a large amount of in-degree and out-degree engagement during #SaludTues Tweetchats. Upon closer examination of our network illustration, it appears that many users are not connected to each other. This is most likely due to the diversity of professional sectors from which #SaludTues users come from. Therefore, we believe #SaludTues chats present an important means of connecting users across disciplines and areas of expertise to amplify important health equity themes. Although #SaludTues participants come from different professions, tweets shared during the chat appear to be part of an echo-chamber, with most users holding similar views to others [53]. However, #SaludTues Tweetchats remain an open platform for debate, information seeking, and raising awareness of policy solutions to promote health equity.

### Lessons Learned

Several lessons can be taken from the *Salud America!* team’s experience with #SaludTues Tweetchats. When seeking cohosts, it is important to look at a potential cohost’s Twitter feed, to determine whether they regularly retweet other users or if their feed is primarily their own content. In addition, it is imperative to determine if they regularly reply to others and if their message is consistent with yours or aligns with your goals and audience, as it can help build partnerships. If you want your cohost to retweet your tweets, you will want someone that already retweets on a regular basis. One might also look at whether the user echoes sentiment, clarifies facts, or provides a unique perspective. Tweetchat hosts may also want an outside perspective to challenge the status quo or may want to avoid confusion and steer clear of conflicting messages. Moreover, it is important to ensure that cohosts are culturally aware and able to discuss health equity issues without victim blaming, stereotypes, or stigmatizing language. It is also best when cohosts are able to share images, graphics, videos, and weblinks as part of their tweets, as these tweets tend to get greater engagement.

### Limitations

There are some limitations to this study. At the platform level, Twitter users are not necessarily representative of the general public. At the individual level, participation in tweetchats depends on internet access and knowledge of how to use Twitter, which could be a potential barrier, even though Latinx people are heavy users of social media. In the analysis of #SaludTues Tweetchats, only tweets using the correct hashtag (#SaludTues) were included. Although social impressions are widely cited as an important metric of a tweet’s reach, they may not fully assess audience engagement, as it is unknown how many followers are active or how much overlap occurs in followers. Therefore, total impressions may appear higher as a result of some Twitter accounts having some of the same followers. We are also unable to directly correlate greater hashtag impressions with increased funneling to the *Salud America!* website or improved self- and collective efficacy or action to address inequity; however, we do expect those outcomes to have occurred. Follow-up surveys of participants of #SaludTues Tweetchats may help elucidate whether participation has increased their self- and collective efficacy for advocacy. Finally, there are several limitations to using Symplur to collect data over large periods. For instance, Symplur only captured the top 1000 users with its stakeholder segmentation tool. Furthermore, Symplur ranks individuals by computing individual variables (eg, replies and retweets), which can be manipulated by excessive tweeting or spamming tools and potentially promote artificially high rankings. Despite these limitations, our experience demonstrates that tweetchats can be a powerful tool for the widespread engagement of audiences to raise exposure to, awareness of, and opportunities for promoting advocacy for health equity. Furthermore, although there are some limitations to using Symplur for data analysis, its ability to quickly capture tweets from a particular hashtag and the ease of using this tool make it practical for use in regularly capturing metrics on health-related tweetchats.

### Conclusions

From our #SaludTues Tweetchat series between 2014 and 2018, we identified key structural elements that we believe helped with the success of these chats. The preparation and planning of these topics in advance while matching those topics to organizational goals was key. In addition, taking steps to secure influential cohosts and additional participation, scripting engaging tweetchat questions, spending time to promote each chat with specialized messaging and graphics, and effectively tweeting in real time during tweetchats are important elements to a successful chat. Archiving tweetchat data for analysis and having access to analytic tools that provide additional insights are necessary for identifying new communities or important networks of users and key influencers participating in #SaludTues.

It is important to note that substantial effort goes into daily digital content curation for the *Salud America!* website, without which tweetchats as a tool to build self-efficacy for advocacy may not be as powerful. Additional research is warranted to demonstrate the impact of tweetchats on particular audiences, networks, website traffic, locations, sentiment, and topics within Latinx health equity, which could augment the true value of using tweetchats to disseminate important health messages and advocacy actions.

## Appendix

Multimedia Appendix 1 Title, cohosts, users, tweets, impressions, and Twitter acquisitions for four #SaludTues Tweetchats in April 2018.

Multimedia Appendix 2 Symplur's stakeholder health care stakeholders based on a user's profile.

## Abbreviations

GA: Google Analytics
SCT: Social Cognitive Theory
