# Supplementary material for: Use of #SaludTues Tweetchats for the Dissemination of Culturally Relevant Information on Latino Health Equity: Exploratory Case Study
Source: JMIR Public Health Surveill. 2021 Mar 1;7(3):e21266. doi: 10.2196/21266 (PMC7961409; doi:10.2196/21266)
Supplement: Multimedia Appendix 1 [file publichealth_v7i3e21266_app1.docx]

| Appendix A: *Title, cohosts, users, tweets, impressions, and Twitter acquisitions for four #SaludTues Tweetchats in April 2018.* | | | | | | | | | | | | | | | | | | |  |
| --- | --- | --- | --- | --- | --- | --- | --- | --- | --- | --- | --- | --- | --- | --- | --- | --- | --- | --- | --- |
| Title | Date | | Cohosts | | Users | Tweets | | | Retweets | Tweets with Media | Tweets with Link | | | Tweets with Mentions | | Impressions | | Twitter Referrals/ Website Users from Twitter | |
| How to Improve Access to Healthy Foods/Drinks | 4/3 | | @Voices4HK @healthyschools @First5LAParents @SugarFreeKidsMD @WVOHCoalition | | 96 | 749 | | | 380 | 276 | 321 | | | 562 | | 6,604,860 | | 42 | |
| Health & Safety at Home for Latino Kids | 4/10 | | @SeguridadConsum @USCPSC | | 27 | 200 | | | 52 | 93 | 41 | | | 108 | | 3,082,590 | | 37 | |
| Climate Changes Health: Transportation & Community Design | 4/17 | | @PublicHealth, @MobilityLabTeam | | 106 | 776 | | | 472 | 191 | 255 | | | 585 | | 11,362,023 | | 42 | |
| Our Kids and the Need for Social and Emotional Learning | 4/24 | | @WINGSforKids @afterschool4all | | 63 | 486 | | | 297 | 201 | 125 | | | 388 | | 5,618,974 | | 75 | |
| **Total** |  |  | | 292 | | | 2211 | 1201 | | 761 | | 742 | 1643 | | 26,668,447 | | 196 | |  |
| Data compiled from Symplur and Google Analytics for chats that took place during the month of April 2018. | | | | | | | | | | | | | | | | | | |  |
