# Supplementary material for: Use of #SaludTues Tweetchats for the Dissemination of Culturally Relevant Information on Latino Health Equity: Exploratory Case Study
Source: JMIR Public Health Surveill. 2021 Mar 1;7(3):e21266. doi: 10.2196/21266 (PMC7961409; doi:10.2196/21266)
Supplement: Multimedia Appendix 2 [file publichealth_v7i3e21266_app2.pdf]

Appendix B. *Symplur's Stakeholder Healthcare Stakeholders Based on a User's Profile*

| **Healthcare Stakeholder** | **Definition** |
|---|---|
| Doctor | Those believed to be licensed, MDs, DOs, PhDs who bill directly for services. Also includes medical residents |
| HCP | Those believed to be other healthcare professionals (i.e., nurses, dietitians, respiratory therapists, nurses, pharmacists, etc.) |
| Patient Advocate | Person who publicly self-identify in their Twitter bio as a patient advocate for a specific disease or condition |
| Caregiver | A professional caregiver or a person who is currently or has been a caregiver of a family member or other closely associated individual |
| Researcher/Academic | Person who is working in the field of health-related research and/or academia. Note: A PhD who does not treat patients falls in this category |
| Journalist/Media | Person whose profession is journalism or other news-related media. Doctors who are editors of journals do not get this label |
| Individual Other Health | Person working in the healthcare industry in a nonclinical role |
| Individual Non-Health | Person not known to be directly working in in the healthcare industry |
| Org. Provider | Inpatient facilities, medical groups, labs, imaging centers, and other outpatient facilities |
| Org. Research/Academic | Accredited schools of higher learning (i.e., universities, colleges, etc.) and healthcare research institutions/centers |
| Org. Government | Government accounts at local, state and national levels |
| Org. Advocacy | An organization focused on a specific set of health issues or medical specialty for the purpose of support, guidance, and education |
| Org. Pharma | All organizations in the pharmaceutical industry |
| Org. MedDevice | All organizations in the medical device industry |
| Org. Media | All organizations whose primary purpose is publishing or broadcasting |
| Org. Other Healthcare | Organizations fulfilling roles within the healthcare industry but not providing direct clinical care |
| Org. Non-Health | All organizations not falling into an established category |
| Spam | Accounts reported to be associated with spam |
| Unknown | Not categorized |

**Notes:**
- When an organization is part of a larger organization, then categorize similarly as to the parent organization (ex. fellowship program part of a hospital: should be Org. Provider. News account from a medical association should be Org. Advocate/Support.)
- Doctors. This includes residents. Medical students should be categorized as "Individual Other Health"

*The definitions and notes are available on Symplur's website.
